# Upregulation of TLRs and IL-6 as a Marker in Human Colorectal Cancer

**DOI:** 10.3390/ijms16010159

**Published:** 2014-12-24

**Authors:** Chien-Chang Lu, Hsing-Chun Kuo, Feng-Sheng Wang, Ming-Huey Jou, Ko-Chao Lee, Jiin-Haur Chuang

**Affiliations:** 1 Division of Colorectal Surgery, Department of Surgery, Chang Gung Memorial Hospital-Kaohsiung Medical Center, Chang Gung University College of Medicine, Kaohsiung 833, Taiwan; E-Mail: kmch4329@gmail.com; 2 Graduate Institute of Clinical Medical Sciences, College of Medicine, Chang Gung University, Taoyuan 330, Taiwan; 3 Department of Nursing, Chang Gung University of Science and Technology, Chiayi 61363, Taiwan; E-Mail: guscsi@gmail.com; 4 Chronic Diseases and Health Promotion Research Center, Chang Gung University of Science and Technology, Chiayi 61363, Taiwan; 5 Research Center for Industry of Human Ecology, Chang Gung University of Science and Technology, Taoyuan 333, Taiwan; 6 Department of Medical Research, Chang Gung Memorial Hospital-Kaohsiung Medical Center, Chang Gung University College of Medicine, Kaohsiung 833, Taiwan; E-Mails: wangfs@ms33.hinet.net (F.-S.W.); chou0131@adm.cgmh.org.tw (M.-H.J.); 7 Department of Surgery, Chang Gung Memorial Hospital-Kaohsiung Medical Center, Chang Gung University College of Medicine, Kaohsiung 833, Taiwan

**Keywords:** Toll-like receptors, real-time PCR, immunohistochemical stain, Western blotting, colorectal cancer

## Abstract

Toll-like receptors (TLRs) not only form an important part of the innate immune system but also serve to activate the adaptive immune system in response to cancer. Real-time PCR; immunohistochemical stain and Western blotting analyses were performed to clarify molecular alterations in colorectal cancer (CRC) patients. We identified Toll-like receptor 1 (*TLR1*), *TLR2*, *TLR4* and *TLR8* gene expression levels and downstream gene, *i.e.*, *interleukin-6* (*IL-6*), *IL-8*, *interferon-α* (*IFN-α*) and myeloid differentiation primary-response protein-88 (*MyD88*), expression levels in CRC patients and in cancer cell lines. CRC tissues have higher *TLR1*, *TLR2*, *TLR4*, *TLR8*, *IL-6* and *IL-8* gene expression levels than do the normal colon mucosa (*p <* 0.05). TLR2 expression varied in different cell types (mucosa and lymphocytes). There was no difference in the *MyD88* and *IFN-α* gene expression levels between cancerous and normal colon mucosa. CRC patients had higher levels of IL-6 (*p* = 0.002) and IL-8 (*p* = 0.038) expression than healthy volunteers did; and higher IL-6 and IL-8 expression was also found to signify a higher risk of recurrence. CL075 (3M002) treatments can reduce the production of IL-8 in different cancer cell lines. The signaling pathway of TLRs in cancer tissue is different from that in normal cells; and is MyD88-independent. Higher expression levels of TLR1, TLR2, TLR 4 and TLR 8 mRNA were related to upregulation inflammatory cytokines *IL-6* and *IL-8* gene expression in tissue and to the upregulation of IL-6 in blood. The concentration of IL-6 in serum can be used as an indicator of the possibility of CRC recurrence. Treatment with 3M002 can reduce IL-6 production *in vitro* and may prevent CRC recurrence. Our findings provide evidence that *TLR1*, *TLR2*, *TLR4* and *TLR8* gene expression induce downstream *IL-6* and *IL-8* gene expression; detection of these expression levels can serve as a CRC marker.

## 1. Introduction

Colorectal cancer (CRC) is the third most common type of cancer and the fourth leading cause of cancer-related death worldwide [1]. In animal models [2,3] and in human patients [4], bacteria are associated with inducing chronic inflammation (or inflammatory bowel disease (IBD)) and colitis-associated colon cancer. The cellular and humoral immune systems have been implicated in the response to tumor antigens in CRC cell lines. The discovery of Toll-like receptors (TLRs) reveals the molecular level of the adjuvants’ role in effective immune potentiation [5,6]. Ten members of the toll-like receptor family have been identified in humans. TLR1 is the first mammalian TLR to have been described and is widely expressed in leukocytes [7]. TLR2 and TLR4 are known to recognize peptidoglycan and lipopolysaccharide (LPS) on the surface of bacteria. The extracellular domain of TLR4 forms a homodimer complex with the MD-2 protein, and it plays a critical role in LPS recognition [8,9]. TLR3, TLR5, TLR7/TLR8 and TLR9 recognize double-stranded RNA, bacterial flagellin, single-stranded RNA and CpG DNA, respectively [10,11]. Among the TLRs, TLR7, TLR8 and TLR9 function inside the endosome [12].

Different TLR ligands have been implicated in various experimental tumor models and are known to play different roles. While some TLRs inhibit tumor progression, others facilitate the evasion of immune surveillance. A number of recent clinical trials have been carried out using TLRs in the treatment of different cancers and other clinical applications [13]. TLR3 is activated by synthetic dsRNA, which induces apoptosis of human breast cancer cells [14,15]. Colitis-associated cancer development can be promoted by numerous TLRs activation [16,17]. Several studies have indicated that TLR4 expression in CRC implies its loss of expression and downregulation, contributing to the metastatic potential of cells [17]. While several observers have suggested that the primary colon-cancer-carrying TLR mutant showed an advanced tumor stage of metastasis, tumor stages were lower at diagnoses [18]. In addition, studies have found that the downstream target of the TLRs signaling pathway, such as Interleukin-6, 8 (IL-6, IL-8), are inflammatory cytokines that play a role in colon cancer. A high level of serum IL-6 correlates with larger tumor size, elevated serum levels and liver metastasis [19]. IL-8 is a proinflammatory CXC chemokine that is highly expressed in cancer cells, endothelial cells, infiltrating neutrophils and tumor-associated macrophages [20]. TLRs’ signaling is initiated by the myeloid differentiation primary-response protein-88 (MyD88) and results in the activation of nuclear factor κB (NF-κB) and mitogen-activated protein kinases (MAPK), which is dependent on MyD88 complexes [21].

In this study, we identified the *TLR1*, *TLR2*, *TLR4* and *TLR8* gene expression in CRC patients and downstream genes, *i.e.*, *IL-6*, *IL-8*, *interferon-α* (*IFN-α*) and *MyD88* expression. The aim was to identify different TLRs and downstream gene expression patterns in normal and cancerous tissues of patients, perhaps leading to the information required for the possible application of one or two genes as a CRC marker based on its gene expression.

## 2. Results

### 2.1. TLR1, TLR2, TLR4, TLR8, MyD88, IFN-α, IL-6 and IL-8 Expression Levels in Normal Mucosa and Colorectal Cancer Tissues from Patients

**Figure 1:**
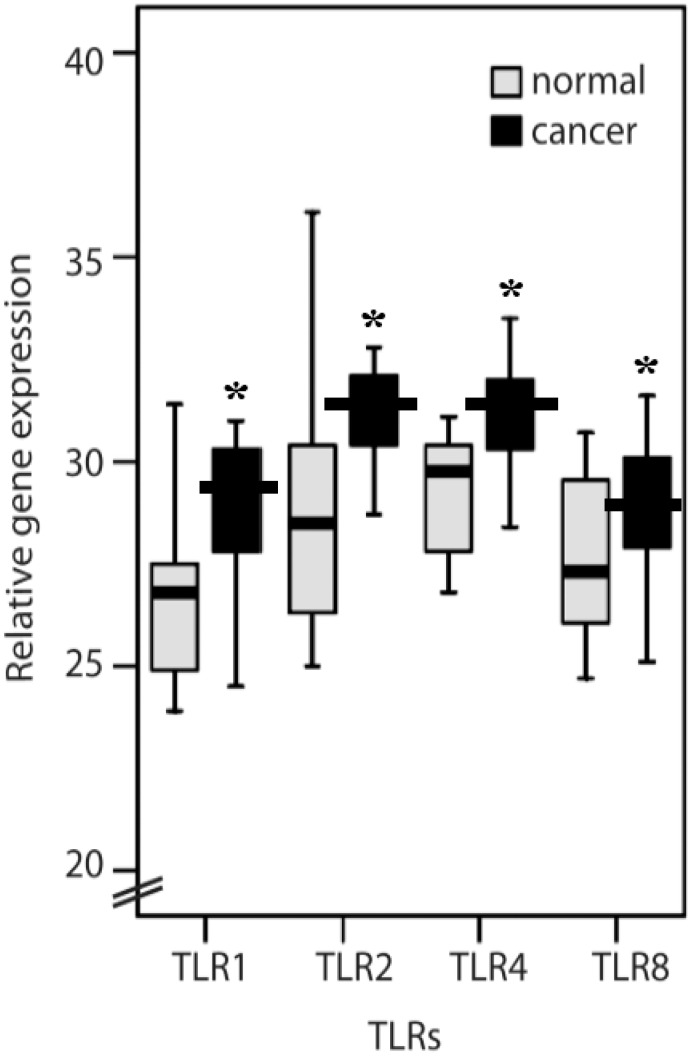
qRT-PCR of Toll-like receptor 1 (*TLR1*), *TLR2*, *TLR4* and *TLR8* gene expression in normal and cancerous tissue (* *p* < 0.05).

**Figure 2:**
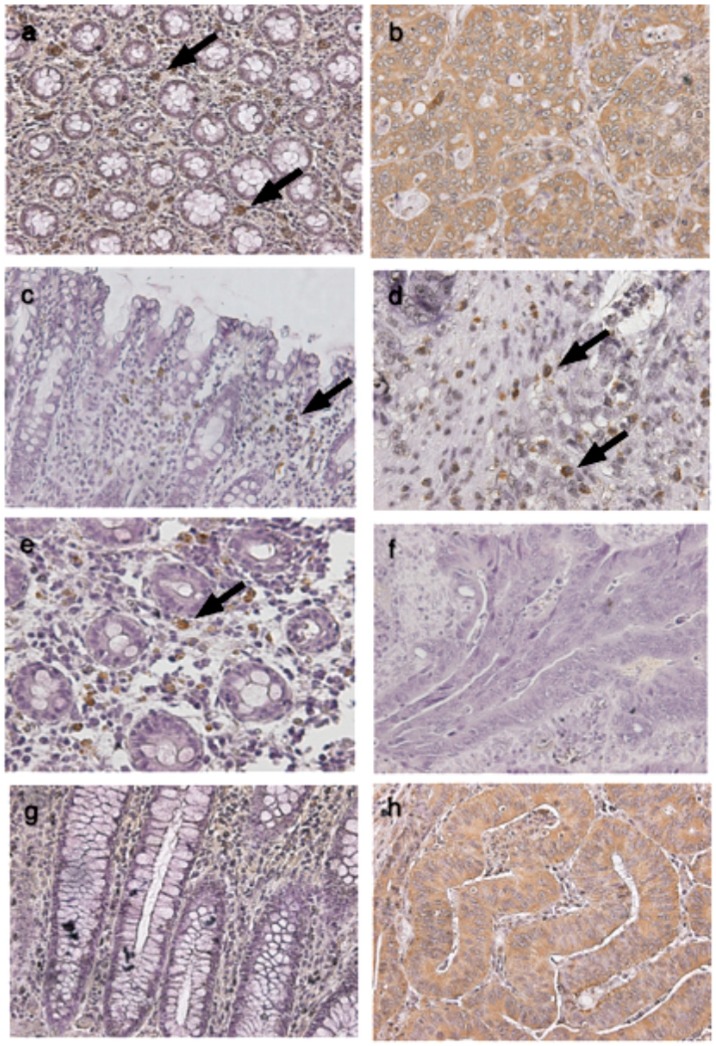
Immunohistochemistry to analyse protein expression of TLR1, TLR2, TLR4 and TLR8 in normal colon mucosa and cancer tissue obtained from the same patient. The representative pictures show that (**a**) TLR1 immunoreactivity is mainly found in inflammatory cells in normal mucosa (black arrows); (**b**) TLR1 is strongly immunoreactive in cancer cells; (**c**) Some TLR2 immunoreactive cells are present in normal mucosa (black arrow); (**d**) A large number of TLR2 immunoreactive tumor-infiltrating cells are present in cancer tissue (black arrows); (**e**) Some TLR4 immunoreactive cells are present in normal mucosa (black arrow); (**f**) TLR4 immunoreactive cells were not present in cancer tissue; (**g**) TLR8 immunoreactive cells were not present in normal mucosa; and (**h**) Strong TLR8 immunoreactivity is present in the cancer cells.

**Figure 3:**
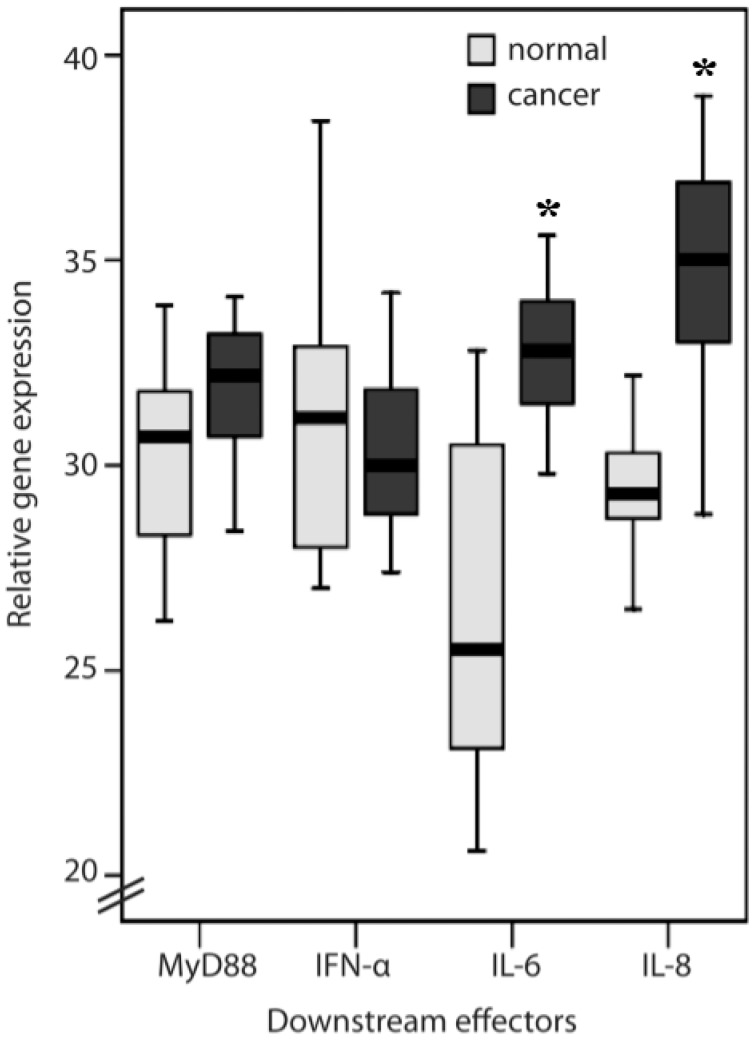
Myeloid differentiation primary-response protein-88 (*MyD88*), *IFN-α*, *IL-6* and *IL-8* gene expression in normal mucosa and colorectal cancer tissues from patients (* *p* < 0.05).

Colorectal cancer tissues have higher *TLR1*, *TLR2*, *TLR4* and *TLR8* (Figure 1) gene expression levels in general than do the normal colon mucosa from the same patient (*p <* 0.05). However, protein expression of TLRs examined in colorectal cancer tissues from patients showed unexpectedly differential results from gene expression assay. There were strong TLR1 and TLR8 immunoreactivities in the cancer cells and in some inflammatory cells in the mucosa. Normal and cancerous tissues showed a significant difference in TLR1 (81.2% and 38.8% of score 1 in normal and cancerous tissue, respectively; 18.7% and 61.1% of score 2 in normal and cancerous tissue, respectively; *p =* 0.015) and TLR8 (81.2% and 33.3% of score 1, as weak, in normal and cancerous tissue, respectively; 18.7% and 66.6% of score 2, as strong, in normal and cancerous tissue, respectively; *p =* 0.006) protein expressions (Figure 2; Table 1). TLR2 and TLR4 immunoreactivity was present mainly in the tumor-infiltrating inflammatory cells (lymphocytes), which were morphologically identical to monocytes/macrophages. A significant difference (75% and 33.3% of score 1 in normal and cancerous tissue, respectively; 18.7% and 66.6% of score 2 in normal and cancerous tissue, respectively; *p =* 0.018) of TLR2 protein expression between normal and cancerous tissues was shown in the lymphocytes (Table 1). In the mucosa, TLR2 protein expression had no difference in either score 1 (62.5% and 83.3% in normal and cancerous tissue, respectively) and score 2 (37.5% and 16.6% in normal and cancerous tissue, respectively) cells of normal and cancerous tissues. Similarly, the expression of TLR4 was mainly in the inflammatory cells of the normal mucosa rather than cancerous tissue (81.2% and 77.7% of score 1 in normal and cancerous tissue, respectively; 18.7% and 22.2% of score 2 in normal and cancerous tissue, respectively; *p =* 0.018; Figure 2; Table 1). However, TLR1 and TLR8 were highly expressed in CRC cells. The related gene expression of *IL-6* and *IL-8* (Figure 3) in cancerous tissues was higher than the normal colon mucosa from the same patient (*p* < 0.05). There was no difference in the *MyD88* and *IFN-α* gene expression between cancerous and normal colon mucosa.

### 2.2. Clinicopathological Features of TLR1, TLR2, TLR4, TLR8, IL-6, IL-8 and MyD88

Table 2 shows the co-distribution of CRC with a high or low *TLR1*, *TLR2*, *TLR4*, *TLR8*, *IL-6*, *IL-8* and *MyD88* gene expressions in relation to cancer and patient characteristics. A total of 11 cancers displayed high expression, and seven cancers showed low expression of TLR1. Nine cancers displayed high expression, and seven cancers showed low expression of TLR2. Four cancers displayed high expression, and 14 cancers showed low expression of TLR4. Twelve cancers displayed high expression and six cancers showed low expression of TLR 8. Eight cancers displayed high expression, and 17 cancers showed low expression of IL-6. Nine cancers displayed high expression, and 16 cancers showed low expression of IL-8. Eleven cancers displayed high expression, and seven cancers showed low expression of MyD88. *TLR1*, *TLR2*, *TLR4*, *TLR8*, *IL-6*, *IL-8* and *MyD88* gene expression had no significant difference between ages of patients, cancer histology and stages of the cancer. Interestingly, low expression of TLR4 was significantly associated with male patients (*p* = 0.045; Table 2); MyD88 expression was related to the tumor site of the colon or rectum (*p* = 0.034; Table 2).

**Table 1:** Intensity of TLR1, 2, 4, 8 immunoreactivity.

Score *	Normal (*n =* 16)	Cancer (*n =* 18)	*p*-Value
TLR1			**0.015**
1	13	7	
2	3	11	
TLR2 (mucosa)			0.163
1	10	15	
2	6	3	
TLR2 (lymphocytes)			**0.018**
1	12	6	
2	4	12	
TLR4			0.571
1	13	14	
2	3	4	
TLR8			**0.006**
1	13	6	
2	3	12	

*n =* number of patients; * Immunoreactivity was scored using a semi-quantitative scoring method; Score 1: immunoreactivity in less than 50% of mucosa, submucosa, and cancer cells (weak); Score 2: immunoreactivity in greater than 50% of the total stained tissues (strong). Bold values indicate *p* < 0.05.

**Table 2:** Clinicopathological features according to TLR1, TLR2, TLR4, TLR8, IL-6, IL-8 and MyD88 expression.

	TLR1			TLR2 (tumor)			TLR4			TLR8			IL-6			IL-8			MyD88		
Clinicopathological Factors	High *n =* 11	Low *n =* 7	*p-*Value	High *n =* 9	Low *n =* 7	*p-*Value	High *n =* 4	Low *n =* 14	*p-*Value	High *n =* 12	Low *n =* 6	*p-*Value	High *n =* 8	Low *n =* 17	*p-*Value	High *n =* 9	Low *n =* 16	*p-*Value	High *n =* 11	Low *n =* 7	*p-*Value
**Age (years)**																					
≥65	7	6	0.308	6	4	0.696	4	9	0.160	8	5	0.457	5	11	0.915	7	9	0.282	6	4	0.059
<65	4	1		3	3		0	5		4	1		3	6		2	7		1	6	
Sex																					
Male	9	3	0.087	6	5	0.838	1	11	**0.045**	8	4	1.0	1	7	0.152	6	11	0.915	5	8	0.682
Female	2	4		3	2		3	3		4	2		7	10		3	5		2	2	
**Tumour Site**																					
colon	4	3	0.783	3	3	0.696	3	4	0.093	5	2	0.732	2	5	0.819	2	5	0.629	5	2	**0.034**
rectum	7	4		6	4		1	10		7	4		6	12		7	11		2	8	
**Histology**																					
Well	0	1	0.387	0	0	0.09	1	0	0.098	0	1	0.339	0	2	0.534	0	2	0.513	0	1	0.155
Moderate	8	5		6	7		3	10		9	4		7	14		8	13		5	9	
Poor/mucinous	3	1		3	0		0	4		3	1		1	1		1	1		2	0	
**Stage**																					
I	1	1	0.985	0	1	0.694	1	1	0.376	1	1	0.896	1	2	0.727	1	2	0.657	0	1	0.285
II	3	2		3	2		2	3		3	2		2	8		5	5		2	3	
III	2	1		2	1		0	3		2	1		1	2		1	2		2	0	
IV	5	3		4	3		1	7		6	2		4	5		2	7		3	6	

IL-6: interleukin-6; IL-8: interleukin-8; MyD88: myeloid differentiation factor 88; TLR: Toll-like receptor. Bold values indicate *p* < 0.05.

### 2.3. High Levels of IL-6 and IL-8 Secretion in CRC Patients Correlated with Higher Risks of Recurrence

**Figure 4:**
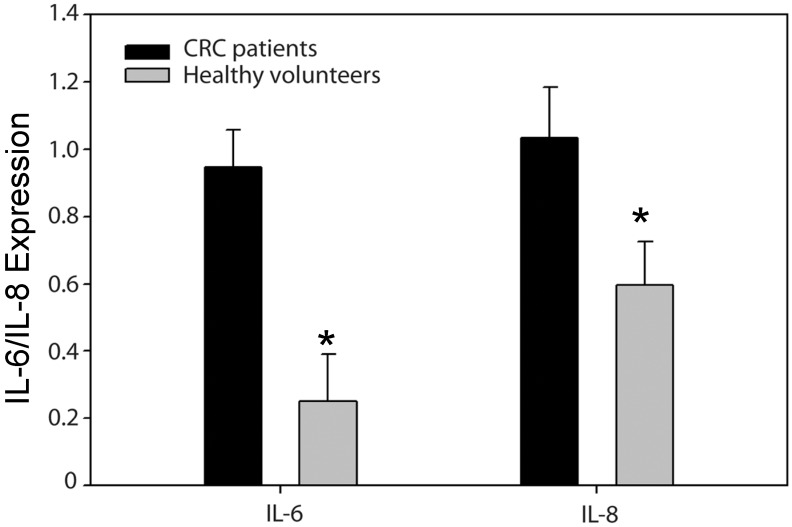
ELISA analysis IL-6 and IL-8 of colorectal cancer (CRC) patients and healthy volunteers (* *p* < 0.05).

**Figure 5:**
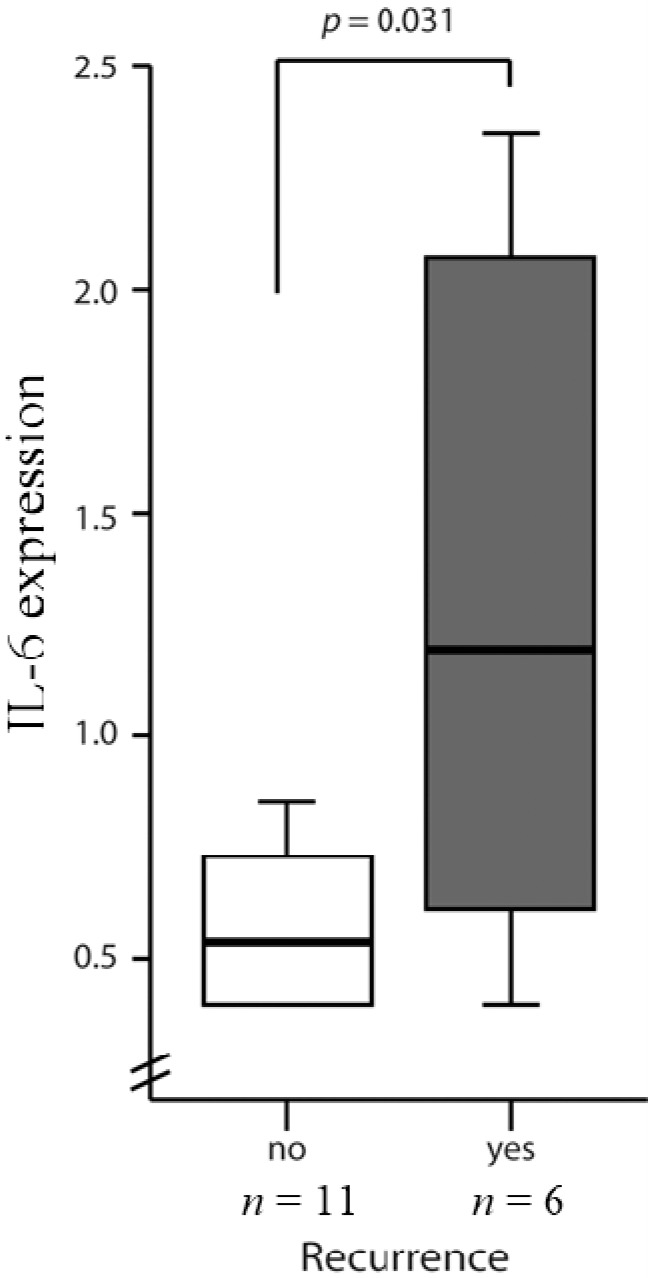
IL-6 levels in Stage II and III CRC patients with and without recurrences. IL-6 ELISA data was classified into two groups using a cutoff value of 10 pg/mL. IL-8 ELISA data was classified into two groups using a cutoff value of 100 pg/mL.

The data from the ELISA showed that CRC patients had higher levels of IL-6 concentration that was adjusted for the age difference (0.9467 ± 0.11 *vs.* 0.2499 ± 0.14; *p* = 0.002). Seventeen of the CRC patients who were in stages II and III had significantly higher *IL-8* gene expression than healthy volunteers, considered to be free of malignancy and inflammatory colon mucosa (log IL-8: 1.035 ± 0.15 *vs.* 0.596 ± 0.13; *p* = 0.038) (Figure 4). There was also a statistically significant difference in the IL-6 values between those with (*n =* 6) and those without (*n =* 11) recurrences (1.3 ± 0.32 *vs.* 0.64 ± 0.11; *p* = 0.031) at the six-month follow-up point (Figure 5). The statistical data also showed a significant difference even when 10 pg/mL of IL-6 was used as a cutoff point (*p =* 0.028). On the other hand, IL-8 ELISA data were classified into two groups using a cutoff value of 100 pg/mL. Five patients had higher IL-8 (>100 pg/mL), and one of them had fecal peritonitis. One was at stage IV with liver metastasis, and the others had recurrences, including two pulmonary metastases and one liver metastasis. This contrasted with patients with low levels of IL-8 (<100 pg/mL) expression who had an uneventful postoperative course and no recurrence at the 36-month follow-up point (*p* = 0.0229).

### 2.4. Release of Proinflammatory Cytokines IL-8 and IL-6 Was Inhibited after 3M002 Treatments

**Figure 6:**
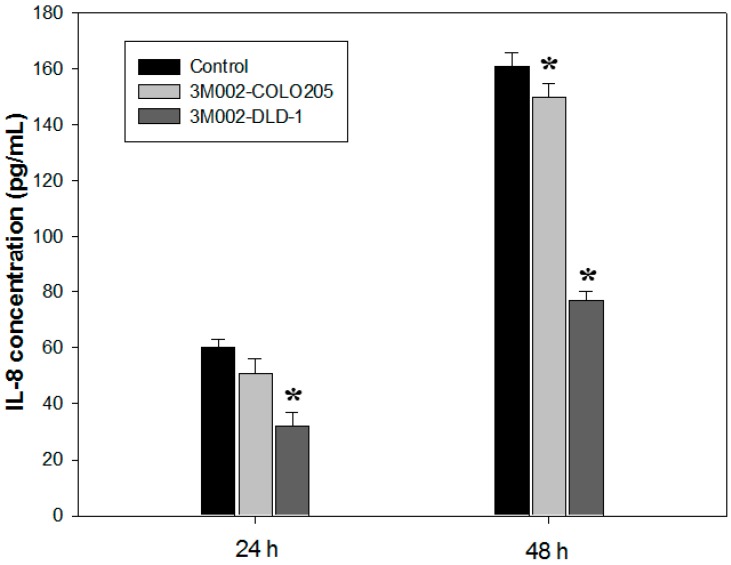
IL-8 expression level of COLO 205 and DLD-1 cell during 3M002 treatment (* *p* < 0.05).

**Figure 7:**
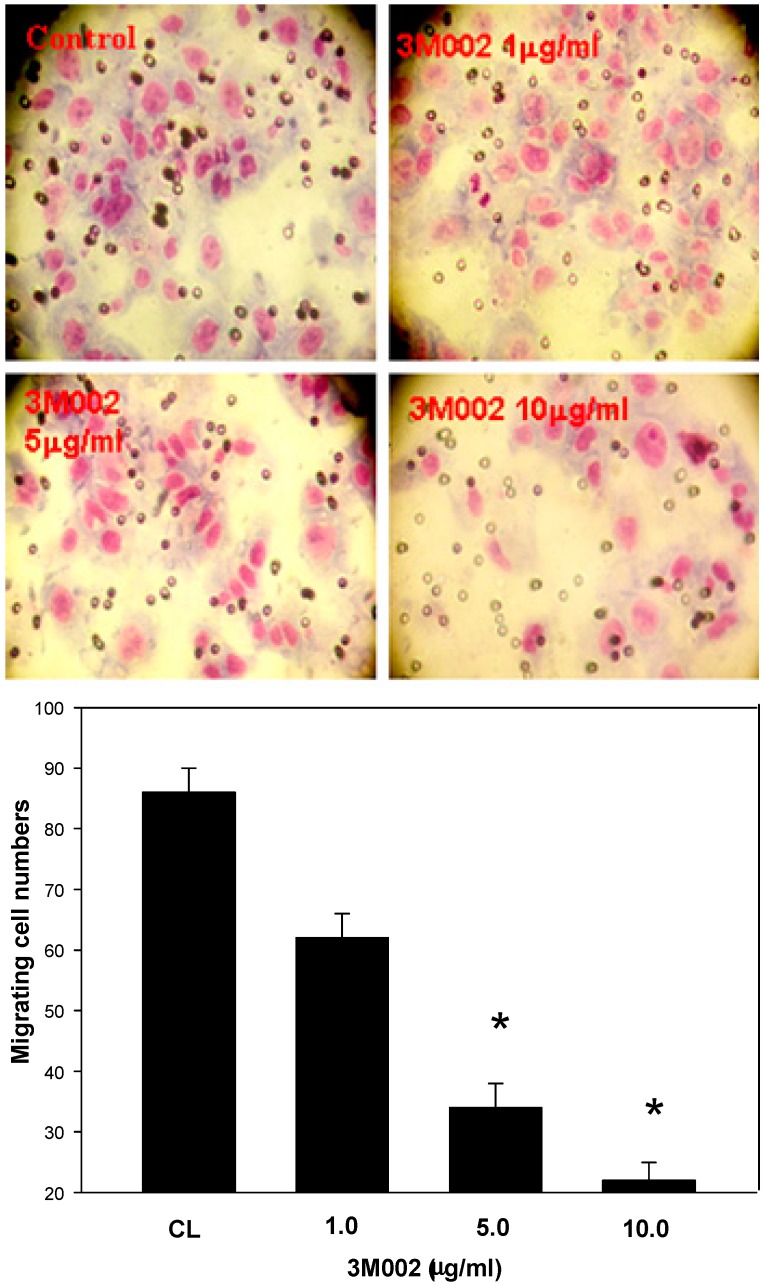
3M002 resulted in a remarkable inhibition of DLD cells migration (* *p* < 0.05).

The IL-8 expression level of the human colon cancer cell (COLO 205 and DLD-1) were inhibited during 3M002 treatment at 24 and 48 h (*p* < 0.05) (Figure 6). A similar result was obtained from the human colorectal carcinoma cell in which the IL-6 expression level of the 3M002-treated cell was higher than that in the untreated cell (data not shown). In addition, the Boyden chamber assay was used to evaluate the *in vitro* migration and invasion effects of 3M002 on DLD-1 cells’ invasiveness. The observations revealed that 3M002 resulted in a remarkable inhibition of CRC cell migration as compared to either treatment alone (Figure 7).

## 3. Discussion

TLR-mediated signaling components such as MyD88 may promote the development of CRC; however, the proinflammatory cytokine IL-1β also uses MyD88 to signal downstream of its receptor [22]. More experiments are needed to demonstrate the relationship between TLR-mediated signaling and CRC formation. In the study by Doan *et al.* [23], the conclusion was that TLR2/4 expression may contribute to colon cancer progression [23]. In our study, the gene expressions of both TLR2 and TLR4 were induced in CRC tissues (Figure 1), while TLR2 and TLR4 levels were shown to have no significant difference between the TMN stages (*p =* 0.694, *n =* 16; *p =* 0.376, *n =* 18; Table 2). On the other hand, TLR2 and TLR4 protein levels shown were focused on normal and cancerous tissues in relation to healthy patients and cancer patients. This indicated that TLR2 and TLR4 were present mainly in the tumor-infiltrating inflammatory cells, not in cancerous tissues (Table 1). Figure 2 shows the immunohistochemistry used to analyze the protein expression of TLR1, TLR2, TLR4 and TLR8 from the same patient. In this study, the different low expression levels of TLR2 and TLR4 in cancerous tissues but high level in the lymphocyte and colon mucosa, which may be due to different TLR-signaling pathways in different types of cells, and then TLR1 and TLR8 were highly expressed in CRC cells. Nevertheless, little is known about its function. In previous study, TLR2/4 agonists induced the production of tumor necrosis factor α (TNFα) and stimulated the expression of inducible nitric oxide synthase (iNOS), therefore causing the tumor cells’ apoptosis [24]. Parenteral TLR2/4 agonists have also been shown to prolong survival in relapsed cancer patients undergoing chemotherapy [25], and reduced expression of TLR4 correlates with increased CRC metastatic progression [17]. However, the absence of *TLR4* ensured by the use of gene knock-out mice can prevent the development of inflammation-induced colorectal tumors. Accordingly, inflammation is an integral step in CRC development; therefore, TLR2/4 function as a suppressor may play a key role in CRC cancer progression in the lymphocytes surrounding the CRC tissue [26,27].

In endothelial cells, TLR4 signaling is inhibited by TLR1 [28]. In cancerous tissue, two very important molecules downstream of TLR4, MyD88 and IFN-α, showed no increase in gene expression levels compared to normal tissue (Figure 3). However, both TLR1 and TLR4 gene expression were induced, and only TLR1 protein was observed in cancerous tissue (Figure 2). Another possibility is that TLR4 is involved in both MyD88-dependent and independent pathways [29], but in CRC, the MyD88-independent pathway is dominant. TLR1 and TLR8 are preferentially expressed on tumor cells, and a high expression of TLR1 and TLR8 (mRNA and protein) was observed in cancer tissues (Figure 2), showing for the first time that TLR1 and TLR8 in humans may be related to CRC. The results from IFN-α and MyD88 in cancer tissues suggested that the TLR8 signaling pathway may be different in cancer tissues from the antiviral responses in which TLR8 induces antiviral responses by MyD88-dependent-producing IFN-α [21].

Downstream effectors of TLRs proinflammatory cytokines include IL6 and IL8 [30]. IL-6, a proinflammatory cytokine of the innate immune system, induces the expansion of T helper cells. Crosstalk between TGFα and IL-6 has been demonstrated in an inflammatory colon cancer model. IL-6 has been shown to play a role in colon cancer *in vivo*, with high serum levels (>12 pg/mL) correlating with larger tumor size and liver metastasis [19]. Moreover, IL-6 has been shown to promote the growth of colon cancer epithelial cells in a cell culture system *in vitro* [31]. Our data demonstrated that elevated IL-6 levels were associated with tumor recurrences but not with stages of the disease. IL-8 functions as a regulatory factor within the tumor microenvironment, and its expression correlates with the angiogenesis, tumorigenicity and metastasis of tumors in numerous xenograft and orthotopic *in vivo* models. Patients with plasma IL-8 levels higher than 100 ng/mL have a poor prognosis. Our findings also support the rationale for targeting IL-8 signaling in numerous other solid tumors (e.g., gastric, pancreatic, melanoma, ovarian, bladder, and prostate).

CL075 (3M002) is a thiazoloquinoline derivative that stimulates TLR7/TLR8 in human peripheral blood mononuclear cells (PBMC), NK cells and T cells. Previous data suggest that the regulation of TLR8 receptors is associated with the immune response, but not tumor cancers [32,33,34]; TLR7/TLR8 have been reported to show potent antitumor activity significantly correlated with stage progression of CRC through the MyD88-dependent pathway [35,36]. In addition, TLR7/TLR8 agonist imiquimod has been successfully used for treatment of various epithelial cutaneous neoplasms [37], and imiquimod 5% cream might represent an alternative topical treatment to surgery in selected cases of Bowen’s disease and squamous skin tumors that exhibit antitumor and antiviral activity through stimulation of both innate and acquired immunity. We investigated the results (Figure 6) that suggest that induction of TLR8 by 3M002 in CRC cancer cell lines can reduce the secretion of IL-6 and IL-8 [38], however, the cellular mechanisms associated with the inhibition of metastasis particularly driven by 3M002 remain to be elucidated.

## 4. Experimental Section

### 4.1. Patients and Specimens

A total of 26 patients with CRC (18 males and 8 females; 66.8 ± 14.2 years of age) who underwent surgery from 2005 to 2006 at the Chang Gung Memorial Hospital-Kaohsiung Medical Center, were investigated in this study. This study was approved by an ethics committee of the Institutional Review Board of the Chang Gung Memorial Hospital. Information on patient demographics (gender and age) and tumor features (anatomical site, histology and TNM stage) was obtained from clinical and pathological records (Table 1). The tumor site was classified as proximal or distal with respect to the splenic flexure. Disease stages were classified according to the criteria proposed by the Standard AJCC (American Joint Committee on Cancer). Eighteen of the samples were obtained as paired normal mucosa/cancer specimens by surgery. Fourteen, four and eight patients were at AJCC stage II, III and IV, respectively. Informed consent was obtained from the patients before surgery. There were seven healthy control volunteers, made up of five men and two women whose mean age was 58.4 ± 8.3 years old. These control volunteers also showed no abnormality, and thus were considered to be free of malignancy and inflammatory colon mucosa.

### 4.2. RNA Isolation and cDNA Synthesis

RNA extraction from the homogenized tissues from colon epithelium (100 mg) using TRIzol Reagent™ (Molecular Research Center, Inc., Cincinnati, OH, USA) was performed according to the manufacturer’s protocol. Briefly, for 100 mg tissue, 1 mL TRIzol regent was added; an additional 200 μL ice-cold chloroform was then used for RNA extraction. Then 500 μL isopropanol was used to precipitate RNA, and RNA was kept in 100 μL DEPC-treated H_2_O. RNA, which had not been used immediately, was kept at −80 °C [21].

Real-time quantitative reverse transcription PCR (qRT-PCR) was performed in order to quantitate the mRNA levels of TLR1, TLR2, TLR4, TLR8, MyD88, IFN-α, IL-6 and IL-8 using the Bio-rad Sequence Detection System (TaqMan; PE Applied Biosystems, Foster City, CA, USA). cDNA was generated from 2 μg of total RNA extracted from fresh tissue using an oligodeoxynucleotide primer (oligo dT15), according to the manufacturer’s instructions (Promega Inc., Madison, WI, USA). PCR was performed in SYBR Green PCR Master Mix (PE Applied Biosystems) containing each of forward and reverse primers, and 30 ng cDNA [39].

### 4.3. Real-Time Quantitative PCR

For reverse-transcription, extracted total RNA as a template was incubated at 42 °C for 30 min in 25 μL reaction volume containing 0.4 mM dNTPs, 5 μL of RT buffer (5×) and 5 U of Moloney Murine Leukemia Virus (M-MLV) reverse transcriptase (Promega Inc.). Reaction buffer 1× containing 2 U (M-MLV) reverse transcriptase (Promega Inc.) and primer pairs, designed according to the consensus sequence of TLRs by using the Primer Express Software version 1.0 (PE Applied Biosystems), were used (Table 3). The PCR conditions were 94 °C, 10 min and 40 cycles at 95 °C for 15 s, 62 °C for 30 s, 72 °C for 15 s; then, 72 °C for 5 min. β-actin was used as an internal control in each sample. RNA isolated from CRC tissues and cDNA were quantified using a spectrophotometer (Shimadzu UV-1201, Kyoto, Japan).

RT-qPCRs were carried out by a GeneAmp 2700 Thermocycler coupled with a GeneAmp^®^ 7000 Sequence Detection System in a 96-well plate. Each well contained 12.5 μL of 2× SYBR^®^ Green Master Mix, 5 μM each of forward and reverse primers, 2 μL DNA and a final volume of 25 μL. The thermal profile for RT-qPCR was 1 cycle at 95 °C for 10 min; 40 cycles at 95 °C for 15 s, 62 °C for 30 s and 72 °C for 15 s [40].

**Table 3:** Primers used in this study. Primer sequences of TLRs and downstream effector molecules including MyD88, IFN-α, IL-6 and IL-8. Primer sequences to amplify β-actin used as a housekeeping control are also indicated.

Name	Sequence	Gene
TLR1-F	5'-CAGCAGCCTCAAGCATGTCTA-3'	*Rat Toll-like receptor-1*
TLR1-R	5'-CAGCCCTAAGACAACAATACAATAGAAGA-3'	
TLR2-F	5'-GCCAGCAGGTTCAGGATGTC-3'	*Human Toll-like receptor-2*
TLR2-R	5'-TGTTCCTGCTGGGAGCTTTC-3'	
TLR4-F	5'-CTTTATTCCCGGTGTGGCCA-3'	*Human Toll-like receptor-4*
TLR4-R	5'-GCAGGGTCTTCTCCACCTTC-3'	
TLR8-F	5'-TTTCCCACCTACCCTCTGGCTT-3'	*Human Toll-like receptor-8*
TLR8-R	5'-TGCTCTGCATGAGGTTGTCGGATGA-3'	
MyD88-F	5'-CCGCCTGTCTCTGTTCTT-3'	*Human MyD88*
MyD88-R	5'-TCCTCCTCAATGCTGGGT-3'	
IFN-α-F	5'-CTATCCCTGTCCTGCATGAGC-3'	*interferon-α*
IFN-α-R	5'-GGGTTGCATCCCAAGCGT-3'	
IL-6-F	5'-GTCAACTCCATCTGCCCTTCAG-3'	*Interleukin-6*
IL-6-R	5'-GGTCTGTTGTGGGTGGTATCCT-3'	
IL-8-F	5'-TCTCTTGGCAGCCTTCCTGA-3'	*Interleukin-8*
IL-8-R	5'-CGCAGTGTGGTCCACTCTCA-3'	
β-actin-F	5'-TCACCCACACTGTGCCCATCTACGA-3'	*Human β-actin*
β-actin-R	5'-CAGCGGAACCGCTCATTGCCAATGG-3'	

### 4.4. Immunohistochemical Studies of TLRs in Cancerous and Normal Tissues

To correlate with the expression of TLRs in cancer and healthy patients, immunohistochemical studies were performed as follows: Tissues from cancer and healthy patients were fixed in 10% formalin and embedded in paraffin. Each two-micron-thick section was cut using a microtome (Leica CM 1900; Leica Microsistemas, Nussloch, Germany) and mounted on a polylysin-coated slide. Deparaffination was performed in xylene and rehydrated in descending grades (30%–100%) of ethanol. To inactivate the endogenous peroxidase activity, 3% hydrogen peroxide was used, followed by microwaving for 10 min in 10 mM citrate buffer to retrieve the antigen. Sections were incubated in 2% normal horse serum (Vector, Burlingame, CA, USA) for 10 min and then followed with anti-TLR1, anti-TLR2, anti-TLR4 or anti-TLR8 antibodies (Santa Cruz Biotechnology Inc., Santa Cruz, CA, USA) incubation (1:50 dilution) at room temperature overnight according to the manufacturer’s instructions. TBS was used to wash the first antibody, and the second antibody was applied and detected using a SuperPicTure™ Polymer detection kit (Zymed Laboratories, South San Francisco, CA, USA) and DAB chromogen (DAKO North America Inc., Carpinteria, CA, USA) were used according to the manufacturer’s instructions. Immunohistochemistry (IHC) stain was quantitative. The presence of cytoplasm stained was scored as positive. The expression of TLRs was quantitatively evaluated using an Olympus CX31 microscope (Olympus, Tokyo, Japan) with an Image-pro Plus medical image analysis system. The digital images were captured using a digital camera (Canon A640; Canon, Tokyo, Japan). The positive area and optical density of the TLRs positive cells were determined by measuring three randomly selected microscopic fields (400× magnification) on each slide. The IHC index was defined as average integral optical density (AIOD) (AIOD = positive area × optical density/total area). The definition of score 1 is immunoreactivity less than 50% of mucosa, submucosa, and cancer cells, as weak, while the definition of score 2 is simply the remaining patients with immunoreactivity greater than 50%, as strong.

### 4.5. Cell Line, Cell Culture and Chemical Treatment

Human colon cancer cell line DLD-1 (CCL-221) and human colorectal carcinoma cell line COLO 205 (CCL-222) were purchased from American Type Culture Collection (ATCC). DLD-1 cells were cultured in RPMI 1640 medium composed of 10% fetal calf serum (FCS) (S0113; Biochrom KG, Berlin, Germany) and 100 units/mL of penicillin (Llorente Laboratories, Madrid, Spain) and incubated at 37 °C with 5% CO_2_. Adhered cells were washed twice with PBS. Then 5 mL of sterile 0.25% trypsin was added and incubated for 5 min at 37 °C in medium containing 5% CO_2_. Then FCS was used to inactivate the trypsin. COLO 205 was cultured in DMEM supplemented with 10% heat-inactivated newborn calf serum at 37 °C in a humidified 5% CO_2_ incubator. DLD-1 and COLO 205 cells were treated with 3M002 (10 μg/mL; 3 M Pharmaceuticals, St. Paul, MN, USA) for varying periods of time (3, 6, 12, and 24 h) and cytokines in secretion were determined by human IL-6 and IL-8 ELISA kit [40]. A Boyden chamber, comprising an upper and a lower compartment, was used to analyze tumor cell migration. Human colon cancer cell lines were allowed to grow as discrete colonies and were treated with 3M002 as previously described [40]. Migration assays were carried out in a 48-well chemotaxis chamber (Neuro Probe Inc., Cabin John, MD, USA). The number of cells that migrated to the lower side of the membrane was then determined.

### 4.6. Enzyme-Linked Immunosorbent Assay (ELISA)

Blood serum were obtained by centrifugation of the blood samples preoperatively from the 26 CRC patients and the 10 healthy volunteers. Cytokine concentrations in the plasma were determined by human IL-6 and IL-8 ELISA kit (R&D Systems, Minneapolis, MN, USA) according to the manufacturer’s instructions [40].

### 4.7. Data Analysis and Evaluation

Real-time fluorescence was measured, and a threshold cycle (*C*_t_) value for each sample was calculated by determining the point at which the fluorescence exceeded a threshold limit, *i.e.*, 10 times above the standard deviation of the baseline. The *C*_t_ values from the samples were plotted on the standard curve, and the copy number was calculated using the Sequence Detection Version 1.6 (PE Applied Biosystems). Each sample was tested in duplicate, and the mean of the two values was used as the copy number of the sample. Samples were defined as negative if the *C*_t_ values exceeded 50 cycles. The *C*_t_ values from 10 healthy volunteers were used as standard and compared to CRC patients; *C*_t_ values from CRC patients higher than those of the average of healthy volunteers were identified as “high” and *vice versa*.

### 4.8. Statistical Analysis

All data are expressed as mean ± standard error. Student *t* test (unpaired, 2-tailed) was used for a comparison of continuous data between the experimental groups. Differences in the distribution of the staining score between the groups were assessed using the Mann-Whitney *U* test. Probability values (*p*) less than 0.05 were considered as statistically significant. Analysis was performed with SPSS software (version 10.0; SPSS, Chicago, IL, USA).

## 5. Conclusions

Several reports focused on the correlation of the TLRs’ signaling pathway and its downstream target gene for the clinical applications as a CRC marker, but the mechanism of this correlation is still unknown. TLRs’ signaling pathway in cancer tissue is different from that in normal cells and is MyD88-independent. Our study was a cross-sectional study, the main purpose of which was to investigate the mechanism of the correlation between the TLRs’ mediated signaling and inflammatory markers in blood samples and mucosa and cancer tissue in the patients. In cancer tissue, the expressions of TLRs (e.g., TLR2) varied in different cell types (mucosa and lymphocytes). Higher levels of expression of TLR1, TLR2, TLR4 and TLR8 mRNA were related to the upregulation of inflammatory cytokines *IL-6* and *IL-8* gene expression in the tissue and the upregulation of IL-6 in the blood. The concentration of IL-6 in the serum can be used as an indicator of the possibility of CRC recurrence. Treatment of 3M002 can reduce the IL-6 and IL-8 production *in vitro* and further studies of the administration of 3M002 *in vivo* are still required to validate these findings to treat CRC. In the future, we may conduct a cohort study with a greater number of tissues and more plasma examination to investigate the association between TLRs’ signaling and inflammatory markers.
